# Experiences in Accessing Treatment Among Females with Schizophrenia: A Qualitative Study from Turkey

**DOI:** 10.3390/healthcare13070721

**Published:** 2025-03-25

**Authors:** Mehmet Cihad Aktaş, Cemile Hürrem Ayhan

**Affiliations:** Faculty of Health Science, Department of Psychiatric Nursing 1, Van Yuzuncu Yil University, Van 65080, Turkey; caktas@yyu.edu.tr

**Keywords:** treatment barriers, schizophrenia, gender

## Abstract

**Background/Objectives**: The experiences of women with schizophrenia in accessing treatment are multifaceted and influenced by a variety of biological, psychological, and social factors. The goal of this study was to explore the experiences of women with schizophrenia in accessing treatment in Turkey. **Methods**: The study was conducted using the phenomenological method, one of the qualitative study methods. In-depth individual interviews were conducted with females with schizophrenia (>18 years) (n = 10). The study data were collected using a personal information form and an open-ended structured interview form in which participants were asked about their views and experiences of accessing treatment. Voice recordings were transcribed, and categories, subthemes and themes were formed. **Results**: Five themes emerged: (1) shadows of obstacles; (2) resistance and adaptation (3) connection and solidarity; (4) unmet needs; and (5) alternative paths. **Conclusions**: This qualitative study on the experiences of accessing treatment among females with schizophrenia in Turkey reveals significant insights into the multifaceted challenges and barriers these women face. The findings indicate that gender-specific factors play a crucial role in shaping treatment experiences and outcomes. Female patients often encounter unique societal pressures, including stigma and domestic coercion, which can influence their treatment adherence and overall mental health outcomes. By addressing the specific barriers faced by women and implementing gender-responsive care strategies, healthcare systems can significantly improve treatment accessibility and outcomes for female patients with schizophrenia in eastern Turkey and beyond.

## 1. Introduction

Globally, 24 million people are affected by schizophrenia, with 90% of them living in developing countries. While both men and women are diagnosed at similar rates, their experiences with recovery are likely to differ [1]. The experiences of women with schizophrenia in accessing treatment are multifaceted and influenced by a variety of biological, psychological, and social factors. Literature emphasized these experiences, focusing on gender differences in treatment responses, the impact of hormonal factors, and the barriers to accessing adequate care [2,3,4].

Research indicates that women with schizophrenia often exhibit different responses to treatment compared to their male counterparts. Females may respond better and more quickly to antipsychotic medications, although inconsistencies exist depending on the specific medications used and individual patient characteristics [5,6]. This gender disparity in treatment response suggests that clinicians should consider sex-specific treatment protocols to optimize therapeutic outcomes for female patients [7]. Furthermore, the role of estrogen in modulating the effects of antipsychotic medications has been highlighted, with evidence suggesting that estrogen may enhance cognitive function and improve symptom management in women [8,9,10].

Despite the potential for effective treatment, women with schizophrenia often face significant barriers in accessing care. These barriers can include societal stigma, lack of awareness about available treatments, and inadequate healthcare resources tailored to their specific needs [11,12]. Additionally, the experiences of stigma and discrimination can disproportionately affect women with schizophrenia, impacting their willingness to seek treatment and adhere to prescribed regimens [13,14]. Research suggests that female patients may experience more severe stigma than their male counterparts, which can exacerbate feelings of isolation and hinder their treatment journey [15]. The intersection of gender and mental health is further complicated by the unique psychosocial challenges faced by women with schizophrenia. Studies have indicated that women are more likely to report personal trauma and struggle with intimate relationships, which can influence their mental health and treatment experiences [16]. These factors necessitate a comprehensive approach to treatment that not only addresses the biological aspects of schizophrenia but also the psychological and social dimensions of the illness [16,17].

Moreover, the cognitive impairments associated with schizophrenia can vary significantly between genders, with females often exhibiting better immediate and delayed memory compared to males [18,19]. This cognitive profile may influence treatment adherence and the overall effectiveness of interventions, suggesting that cognitive assessments should be integrated into treatment planning for women with schizophrenia [18,19].

In summary, the experiences of women with schizophrenia in accessing treatment are shaped by a complex interplay of biological, psychological, and social factors. Gender differences in treatment response, the influence of hormonal changes, and the impact of stigma and discrimination all play critical roles in shaping these experiences. Considering the gender differences in the clinical presentation, treatment, and access to treatment of schizophrenia, this study phenomenologically investigates the treatment access experiences of female schizophrenia patients in eastern Turkey, a conservative and Islamic society. This investigation addressed the following specific research question: (1) What do female schizophrenia patients experience when accessing treatment? The findings are intended to inform improvements in developing gender-sensitive treatment protocols and addressing the barriers that hinder access to care for women with schizophrenia. This study is one of the few qualitative studies that examine the experiences of women diagnosed with schizophrenia in accessing treatment in Turkey. In the existing literature, the barriers women face in psychiatric treatment, the influence of social dynamics, and the structural challenges within the healthcare system have been explored to a limited extent. This research addresses this gap by highlighting the unique experiences of women in their treatment journeys. The findings contribute to making mental health services more responsive to women’s needs and provide insights for policymakers. Additionally, this study aims to guide future research by informing strategies to improve women’s access to mental health services.

## 2. Materials and Methods

### 2.1. Study Design

This study employs a qualitative design to explore the treatment access experiences of women with schizophrenia in eastern Turkey. Specifically, a phenomenological approach was adopted, guided by Colaizzi’s (1978) phenomenological analysis method [20]. This approach allowed for a deep understanding of participants’ personal experiences, perceptions, and meanings regarding their access to treatment. The study is grounded in a constructivist paradigm, which asserts that reality is socially constructed and shaped by individual experiences. Within this paradigm, the phenomenological approach was used to explore the lived experiences of participants [21,22]. Colaizzi’s (1978) phenomenological analysis method was utilized to interpret the data, helping to identify both explicit and implicit meanings in participants’ narratives [20]. Additionally, Goffman’s (1963) Stigma Theory provided a conceptual lens to understand how societal perceptions influence participants’ help-seeking behaviors and treatment adherence [23]. This theoretical framework is particularly relevant in the context of healthcare access and utilization, especially for individuals with schizophrenia, in conservative cultural settings. By integrating this perspective, the study aims to explore how stigma manifests at the individual, familial, and institutional levels, shaping the treatment trajectories of women with schizophrenia. The qualitative design is well suited to investigate complex, context-dependent phenomena such as the ways in which women with schizophrenia access, utilize, interpret, and interact with treatment in a culturally specific context like eastern Turkey. This approach allows for an in-depth examination of patient perspectives, which are influenced by various factors, including socioeconomic constraints, healthcare service accessibility, and cultural or linguistic diversity.

This design limited data collection to a single time point, offering a snapshot of participants’ knowledge and experiences at a specific moment in their treatment journey. This methodology aligns with the study’s objective to generate knowledge that can inform patient-centered treatments and improve the quality of schizophrenia care in similar cultural contexts. By positioning the study within a constructivist paradigm and applying a stigma theory framework, this research provides a nuanced understanding of the barriers and facilitators affecting treatment access among women with schizophrenia in a culturally specific context.

### 2.2. Study Setting

The study was conducted at an outpatient psychiatry clinic affiliated with Van Yuzuncu Yil University Dursun Odabas Medicine Center in the Van located in Eastern Turkey. This facility provides inpatient and outpatient services to patients with severe mental disorders and provides follow-up and treatment. The data were collected between 10 September–30 December 2023. The center was chosen because it serves as a primary healthcare provider in a region where access to mental health services is often limited, especially for women facing multiple social and cultural barriers. Additionally, the center’s patient demographic includes individuals who represent a wide range of treatment experiences, including those in the acute and long-term stages of schizophrenia, making it an ideal setting for examining diverse treatment access experiences. The center’s clinical staff is experienced in working with patients from diverse backgrounds, and its service structure is well-equipped to capture the complexities of accessing and utilizing schizophrenia treatment in a conservative, Islamic context. By selecting this center, the study aimed to ensure that the experiences and perspectives gathered reflect the real-world challenges and realities faced by individuals in similar socio-cultural settings. Access to the field was facilitated by nursing staff and healthcare workers, who played a crucial role in obtaining permission to conduct the study. The gatekeeper, along with the nurses and other staff members, assisted in recruiting participants and providing logistical support throughout the data collection process. This collaborative effort ensured smooth access to the study population and facilitated the engagement of participants.

### 2.3. Participants

A total of 10 females with schizophrenia participated in the study. The demographic characteristics of the participants, including age, marital status, educational status, economic status, and having a child are summarized in Table 1.

The participants consisted of 70% single and all of them were unemployed. The mean age of the participants was 41.40 (min: 25, max: 51). This distribution provides insight into the demographics of female schizophrenia patients at the outpatient clinic affiliated with Van Yuzuncu Yil University Dursun Odabas Medicine Center in this study.

#### 2.3.1. Inclusion Criteria

- Being over 18 years of age.
- Being Woman.
- Diagnosed with schizophrenia according to the DSM 5 or ICD 11,
- Being open to communication and cooperation.
- Individuals who were who were registered and followed up at CMHC-affiliated SBU Van Education and Research Hospital.
- Patients who were physically and clinically stable enough to participate in interviews.

#### 2.3.2. Exclusion Criteria

- The participant had difficulties understanding both written and verbal information, as individuals with limited literacy.
- Having comorbid psychiatric disorders.

The exclusion of participants with comorbid psychiatric disorders was a deliberate methodological choice to ensure that the study specifically focused on the experiences of women diagnosed solely with schizophrenia. Given that comorbid conditions can introduce additional psychological and clinical complexities, their inclusion might have led to variations that could confound the interpretation of schizophrenia-specific lived experiences. Furthermore, ensuring clinical stability was a key inclusion criterion, and the presence of additional psychiatric disorders could have influenced symptomatology, treatment responses, and overall experiences in ways that were beyond the scope of this study. While this approach enhances internal validity by maintaining a more homogenous sample, it is acknowledged that it may limit the generalizability of the findings. Future research should consider exploring the experiences of individuals with schizophrenia and comorbid psychiatric disorders to provide a more comprehensive understanding of this population.

### 2.4. Participant Selection

The participants consisted of 10 women diagnosed with schizophrenia, selected through purposive participant selection. Individual in-depth interviews were conducted to collect data. In qualitative research, particularly in phenomenological studies, achieving data saturation is crucial for determining the adequacy of the number of participants. Data saturation refers to the point at which no new codes, categories, or themes emerge from additional data collection [18]. In this study, data saturation was observed during the 7th interview, when no new codes or themes emerged, and it was confirmed in the 9th interview. To ensure saturation, an additional interview (10th participant) was conducted, but no new insights were obtained. Given the homogeneity of the sample and the depth of the interviews, data saturation was deemed sufficient, and data collection was finalized with 10 participants. Previous studies suggest that phenomenological research typically requires 6 to 15 participants to achieve saturation [15,24,25], supporting the adequacy of the sample size in this study. Furthermore, as the participant group was relatively homogenous, fewer interviews were required to capture the depth of experiences. Considering the richness of the data and the depth of analysis, 10 participants were deemed sufficient to address the research questions and achieve theoretical saturation. The selection process targeted women with schizophrenia who met the inclusion criteria with the help of nursing staff and healthcare workers at the outpatient clinic affiliated with Van Yuzuncu Yil University Dursun Odabas Medicine Center who identified eligible participants and introduced them to the study. Healthcare providers at the hospital identified eligible participants based on age (≥18 years), clinical stability, and inclusion criteria. Once identified, eligible participants were approached by the researcher, who explained the study in detail, provided information sheets, and obtained written informed consent.

### 2.5. Data Collection

#### 2.5.1. Instruments

The data were collected via individual in-depth interviews. In-depth interviews were performed using a tailored interview guide for this research. In-depth interviews were utilized to provide participants with opportunities to express their unique experiences while focusing on key themes relevant to the study’s aims. The interview guide was carefully designed to encompass the following domains: (1) participants’ memories and interpretations regarding getting treatment; (2) circumstances encountered during treatment access; (3) subsequent guidance related to treatment access; (4) individual experiences of treatment access and its perceived effects on their daily lives and quality of life; and (5) comprehension and accessibility of management and referral pathways in the event of symptom appearance. The interview guide was developed through a systematic process to ensure alignment with the research objectives and theoretical framework. Initially, a literature review was conducted to identify key domains relevant to the experiences of women diagnosed with schizophrenia. Expert consultations with psychiatrists, and psychiatric nurses were conducted to refine the thematic areas and ensure their relevance. Based on these insights, a preliminary set of open-ended questions was developed, focusing on participants’ lived experiences, emotional and cognitive responses, coping mechanisms, and interactions with healthcare professionals. To validate the interview guide, a pilot study was conducted with two participants who met the inclusion criteria but were not included in the final sample. Their feedback, along with expert review, was used to refine the wording of questions, ensuring clarity and appropriateness. Furthermore, questions were structured to be non-directive, allowing participants to express their experiences freely without leading them toward specific responses.

To maintain consistency across interviews and minimize potential interviewer bias, several strategies were employed:

1. Standardized Interview Protocol: The same semi-structured interview guide was used for all participants to ensure uniformity in questioning. However, flexibility was maintained to allow participants to elaborate on their unique experiences.
2. Interviewer Training: The primary interviewer underwent training in qualitative interviewing techniques, emphasizing active listening, neutral questioning, and avoidance of leading questions. The training also included mock interviews, where feedback was provided on tone, phrasing, and interaction style.
3. Reflexivity and Researcher Bias Management: The interviewer maintained a reflexive journal throughout the data collection process to document personal reflections, potential biases, and any challenges encountered. This helped enhance self-awareness and ensure an objective approach during the interviews.
4. Member Checking and Peer Debriefing: To further minimize bias, after a subset of interviews, peer debriefing sessions were conducted with other researchers to discuss emerging themes and potential areas of interviewer influence. Additionally, participants were given the opportunity to review and confirm their responses (member checking) to ensure that their experiences were accurately captured.
5. Consistent Probing Techniques: When clarification was needed, the interviewer used neutral prompts such as “Can you tell me more about that?” or “How did that make you feel?” instead of directive or suggestive phrasing that could lead participants toward a particular response.

#### 2.5.2. Procedure

Date: 12 May 2023 Institutional permission was also granted by Van Yuzuncu Yil University Dursun Odabas Medicine Center (Protocol: 412348, Date: 6 September 2023). All ethical considerations for research on human subjects, described in detail later, were adhered to. Second, the interviews were conducted in private rooms within the hospital to ensure confidentiality and minimize interruptions. The interviewer and the participant were alone during each interview. The interviews were conducted in either Turkish or Kurdish, depending on the participant’s preference, to ensure that participants could express themselves in the language they felt most comfortable with. All interviews were audio-recorded and transcribed verbatim in their original language before proceeding with an analysis. For participants who were interviewed in Kurdish, a forward-translation process was conducted, where the transcribed texts were translated into Turkish by a bilingual professional fluent in both Turkish and Kurdish, with expertise in psychiatric terminology. To ensure accuracy and maintain conceptual equivalence, a review process was implemented, where an independent bilingual researcher cross-checked a subset of the translated transcripts against the original audio recordings. Any discrepancies were discussed and resolved to maintain consistency. Thematic analysis was conducted in Turkish, as all research team members were fluent in Turkish. Given that the primary goal was to retain the cultural and contextual integrity of participants’ narratives, a strict conceptual translation approach was prioritized over literal translation. While a full back-translation process was not performed, peer debriefing sessions were conducted with bilingual experts to ensure that the key themes accurately reflected participants’ intended meanings. This approach allowed for both linguistic accuracy and cultural sensitivity, minimizing potential distortions that could arise from direct translation while maintaining the reliability of the data.

Before starting the interview, each participant was informed about using the voice recorder, and their written and verbal consent was obtained. Points considered very important were written down separately, and each in-depth interview lasted approximately 29–43 min. A single researcher interviewed all participants, and the interviews were transcribed on the same day. Recordings were securely saved on a password-protected device available exclusively to the research team.

### 2.6. Data Analysis

In the data analysis, qualitative content analysis was used to create themes and categories within the scope of the research. With this analysis method, the visible content obtained from the participants’ statements and the hidden content underlying the verbal expressions with the researcher’s interpretation were analyzed [26]. Colaizzi’s (1978) phenomenological analysis steps were used for data analysis [20]. (Authors’ work throughout the reporting is indicated by their initials).

While analyzing the data, the following steps were followed in order.

1. Transcription: The interviews on the voice recorder were transcribed into written text (M.C.A.).

2. Familiarization with the Data: First, the transcripts were read repeatedly to understand the participants’ experiences, and short notes were taken (M.C.A.; C.H.A).

3. Open Coding: The transcripts were processed line by line using relevant open coding and content labeling (M.C.A.; C.H.A).

4. Code Categorization: The codes were grouped into categories and subcategories based on conceptual similarities and differences (M.C.A.; C.H.A.). For example, the codes “discrimination”, and “negative perceptions of mental health” were grouped under the category “the impact of stigma”, which was later integrated into the overarching theme “Shadows of Obstacles.” Similarly, the codes *“relocating for treatment access”* and *“strong commitment to recovery”* were grouped under the category *“Mobility for Access”*, forming part of the theme *“Resistance and Adaptation.”*

5. Theme Development: Categories were synthesized into broader themes by identifying underlying patterns across participants’ narratives. The initial themes were reviewed by a psychiatrist to ensure coherence with clinical findings.

6. Consensus and Refinement: The themes were discussed among researchers until a consensus was reached, ensuring alignment with the research objectives (M.C.A.; C.H.A.). The conceptual structure of the themes was further refined through iterative discussions.

7. Member Checking: Participants were contacted via phone and provided with a summary of the findings. Their feedback was incorporated into the final analysis where necessary (C.H.A.). Participants were provided with a summary of the findings through phone, and their feedback was incorporated into the final analysis, where necessary. To ensure the trustworthiness of the data analysis, the following strategies were applied:

Researcher Triangulation: Multiple researchers (M.C.A. and C.H.A.) independently coded the data. The initial coding results were compared, and discrepancies were discussed until a consensus was reached. This approach minimized individual biases and enhanced the credibility of the findings.
Reflexivity: To minimize researcher bias, a reflexive journal was maintained, where researchers noted their assumptions and reflections throughout the analysis process.
Peer Debriefing: To ensure the credibility of the findings, peer debriefing sessions were conducted with an independent qualitative researcher, who reviewed the themes and provided feedback on their accuracy.

By implementing these techniques, the study aimed to ensure a rigorous and transparent analytical process while accurately reflecting the participants’ experiences.

### 2.7. Ethical Considerations

This study was conducted in full accordance with ethical guidelines to protect participants’ rights, ensure anonymity, and preserve the integrity of the research process. Ethical issues were organized according to the concepts of autonomy, kindness, non-maleficence, beneficence, and justice, which are fundamental to ethical research in healthcare environments. Ethical consent was gained from the Van Yuzuncu Yil University Non-Clinical Research Ethics Committee (Protocol No: 2023/05-26 Date: 12 May 2023), along with institutional permission from Van Yuzuncu Yil University Dursun Odabaş Medicine Center (Protocol: 412348, Date: 6 September 2023) to conduct on-site interviews.

Participants were granted informed permission after being thoroughly briefed on the study’s objectives, their entitlement to withdraw at any moment, and the confidentiality of their replies. Consent forms were signed, and verbal consent was also verified for individuals with restricted literacy. Participation was completely optional, and participants were guaranteed that non-participation or withdrawal would not influence their treatment. To ensure anonymity, pseudonyms were employed in transcripts, and all data were securely kept on password-protected devices accessible solely to the research team. Audio recordings and transcripts were maintained separately from identifying information, with all data designated for secure devastation, in accordance with institutional rules at the study’s conclusion. When participants encountered emotional distress when conducting interviews, on-site counseling help was provided. During the interviews, emotional distress was observed in 2 participants. In such cases, the interview was temporarily paused, and participants were given the option to take a break or discontinue their participation. Those who required further support were referred to an on-site counselor, and appropriate psychological support was provided. All participants were informed about these support options before the interviews. This ethical framework, prioritizing respect, autonomy, and secrecy guaranteed that the study was executed responsibly and adhered to the principles of beneficence and non-maleficence.

### 2.8. Rigor and Trustworthiness

To ensure rigor and trustworthiness, including the pilot study, this study employed several strategies grounded in credibility, dependability, transferability, and confirmability. To ensure the consistency of the study, all data were collected by a single researcher (consistency). The participants’ statements were given as they were in the findings, without the researcher’s comments. In addition, the fact that the sample group was selected by purposive sampling strengthens the transferability of the study (transferability). A psychiatrist checked the conformity of the research findings with the themes. The researcher’s interpretations were verified by other researchers through discussion (confirmability). The findings were obtained through individual in-depth interviews. Each participant was given enough time to express their thoughts and feelings. All interviewed women were contacted by phone and questioned about the appropriateness of the emerging themes, and all participants confirmed the results (credibility).

### 2.9. Data Management

This study’s data management emphasized security, organization, and compliance with ethical standards [27]. All interviews were audio-recorded with participant approval and saved on a password-protected device accessible solely to the research team. Interview recordings were transcribed verbatim and anonymized by substituting participant identities with pseudonyms to ensure confidentiality. Transcripts and coded data were securely kept in encrypted digital files, with backup versions maintained on institutional secure servers in accordance with university data security standards. Physical papers, such as permission forms, were secured in a locked cabinet apart from the electronic data. Access to the data during the study was restricted solely to approved members of the research team, therefore assuring the secure and appropriate management of sensitive information. Following the conclusion of the research, all data will be preserved for a designated duration of two years in accordance with the university’s data retention policies and subsequently disposed of safely, with digital files permanently erased and physical papers shredded. This data management procedure guarantees participant confidentiality and adheres to ethical norms for data integrity and security in qualitative research.

## 3. Results

### 3.1. Thematic Analysis Findings

The thematic analysis of participant responses identified several key themes related to the experiences of female schizophrenia patients regarding accessing treatment. Each theme provides insight into specific aspects of content and their experiences with accessing treatment.

#### 3.1.1. Theme 1: Shadows of Obstacles

This theme consisted of three subthemes such as the impact of stigma, clinical barriers, and access barriers. This theme highlights the problems experienced by female patients with schizophrenia in accessing treatment.

The subtheme of the impact of stigma highlights that participants are marginalized and looked down upon by their surroundings due to their illness. People exhibit negative attitudes towards them, and some individuals in their environment advocate for discontinuing medication based on misconceptions about psychiatric illnesses and treatments. It can be argued that environmental challenges create barriers and negative motivations for women diagnosed with schizophrenia in their pursuit of treatment. The current study indicated that the negative attitudes exhibited by the environment regarding medication use hinder women’s ability to access and maintain treatment. The participants’ statements are as follows:

Participant 6: “They told me I was disabled, that I was insane. People’s stares bothered me. They looked at me with disdain. They said I was not human, that I was different, that I could do nothing. My family, friends, and relatives said these things. My uncle told me to die…”

Participant 9: “People around me were saying, ‘Don’t take medication; it will make you worse, and you’ll gain a lot of weight because of these medications.”

The subtheme of clinical barriers highlights obstacles such as compulsory hospitalization, the restrictive nature of psychiatric services, the stigmatizing attitudes of the treatment team, and the inability to establish therapeutic communication with the treatment team at the desired level. This situation creates challenges for women diagnosed with schizophrenia in accessing treatment. Another factor that adversely impacts these women is the compulsion to remain in the hospital, which makes participants uncomfortable. Additionally, the feeling of not being understood by the doctor they consult for treatment further delays the initiation of the treatment process. The participants’ statements are as follows:

Participant 5: “…on the one hand I wanted to get treatment, but I didn’t want to be hospitalized. Then they took me by force.”

Participant 8: “I couldn’t find a doctor like I wanted. I thought they didn’t understand me. I could feel the way people treated me…”

The subtheme of access barriers highlights the challenges faced by women living in villages or districts without mental health specialists, financial constraints such as the absence of social security, and issues like a lack of awareness about their condition. These challenges are linked to financial limitations, geographical distance, and insufficient information. Additionally, the absence of social security complicates access to costly treatments. Another factor hindering treatment access is that some participants lack knowledge about their illness or are unsure which doctor to consult. Furthermore, family members often prevent women diagnosed with schizophrenia from visiting the community mental health center (CMHC), which offers rehabilitation and treatment follow-up, posing another obstacle to effective treatment. The participants’ statements are as follows:

Participant 10: “When I fell ill, my family took me to the hospital, but afterward, I wanted to go to the CMCH, yet my family wouldn’t allow me… I faced financial difficulties.”

Participant 1: “…there were no doctors available when my illness began; it was challenging to find a doctor since we lived in a village. My father took me to Erzurum province to see a doctor. That’s why my treatment started late…”

Participant 2: “I didn’t know what my illness was… they claimed I was possessed by demons… so I didn’t even know which doctor to consult…”

Participant 5: “I couldn’t begin treatment because my insurance had expired. They said that examinations and hospitalization were very costly, so we had to wait until I could renew my insurance.”

#### 3.1.2. Theme 2: Resistance and Adaptation

This theme emphasizes the positive responses of participants in managing the challenges they encounter, such as relocating to facilitate easier access to treatment and demonstrating a strong commitment to recovery. It also addresses the negative responses, including discontinuing medication and remaining homebound due to the impact of stigma. This theme consisted of mobility for access, withdrawal and autonomy, and social isolation subthemes.

The subtheme of mobility for access highlights that women residing far from the treatment center relocate to the city center to continue their care. This situation reflects the women’s strong motivation for treatment and the support they receive from their families. The participants demonstrate eagerness for treatment and possess a high level of awareness. The participants’ statements are as follows:

Participant 1: “We moved so that we can go to the doctor… so I could receive treatment more easily”.

The subtheme of withdrawal and autonomy highlights the stigmatization of psychiatric medication use by society and the resulting withdrawal of female schizophrenia patients from their prescribed treatments. For insight to develop in psychotic disorders, symptoms must be managed, and medication is essential for this. Ceasing medication may lead to diminished insight and a belief that treatment is unnecessary due to symptoms. This scenario is viewed negatively regarding access to treatment. Participants are also cognizant of this adverse situation. The participants’ statements are as follows:

Participant 4: “…some of my relatives were criticizing me a lot, saying, ‘this person is sick, he is on medication’… I stopped taking my medication because I couldn’t accept what they were saying.”

Participant 9: “…I initially stopped taking my medication. Then, when I noticed I was getting worse, I stopped listening to anyone…”

The subtheme of social isolation highlights that the participants interacted with others as little as possible due to the stigma associated with their illness, choosing to isolate themselves to shield themselves against these negative attitudes. The participants’ statements are as follows:

Participant 5: “I was always alone at home; I didn’t go out. I spent time with myself. I felt uncomfortable when guests visited, so I would lock myself in my room.”

#### 3.1.3. Theme 3: Connection and Solidarity

This theme highlights both the importance of individual factors including self-motivation and family support and institutional factors including free access to care and the availability of CMCH for women with schizophrenia to obtain treatment. This theme consisted of three subthemes such as self-motivation, family support, and support from healthcare institutions.

The subtheme of self-motivation highlights women’s desire to regain the functionality they experienced in their pre-disease lives. Women diagnosed with schizophrenia are particularly adversely affected by the decline in their functionality. The aspiration to return to their previous state was recognized as a significant source of motivation for pursuing and maintaining treatment. Some participants demonstrated high awareness and indicated that they utilized their self-dynamics for recovery. A determined attitude towards recovery proved effective in the treatment processes of the participants. The participants’ statements are as follows:

Participant 1: “…I wanted to return to my old self. I wanted to be able to work and sleep like I used to. I knew that this would happen by focusing on my treatment...”

Participants 8: “…I coped on my own. No one helped. I decided; I said, I won’t do this, I will fix myself, and I did what I said...”

The subtheme of family support highlights the significance of the family’s supportive and protective attitude before and during the treatment process in accessing care. The participants’ statements are as follows:

Participant 1: “The support of my brother and father was crucial to me. They consistently took me to the doctor. I couldn’t have done it without them.”

Participant 3: “I receive support from my brother and aunt-in-law. Their children interfere with me because of my illness, but they prevent that. They assist me in taking my medication. My aunt helps me bathe.”

Participant 6: “My mother was my greatest support. When people around me said hurtful things, she was there for me, encouraging me not to care… She aided me with my treatment. She took me to the hospital, provided my medication, and helped with my care. She supported me emotionally.”

The subtheme support from healthcare institutions highlights that the existence of a CMHC, where rehabilitation services are offered, follow-ups and activities for patients are conducted, the provision of free treatment services by the state, and the fact that these patients have social security, facilitates access to treatment. Indeed, some participants noted that the absence of social security hindered their access to treatment. The participants’ statements are as follows:

Participant 7: “My brother assists me when I go to CMHC. It’s wonderful to have that place; at least I can engage in something and keep myself occupied. My family doesn’t have to manage me too much”

Participant 9: “…thank God the state offers free treatment; otherwise, no one would be able to receive care. Our financial situation is clear anyway; it was impossible for us to bear this much burden.

#### 3.1.4. Theme 4: Unmet Needs

The theme of unmet needs highlights the requirements of female schizophrenia patients in obtaining treatment and comprises three subthemes: the need for family support, the need for stigma reduction and emotional support, and the need for financial assistance. It can be stated that women diagnosed with schizophrenia have significant needs in accessing and maintaining treatment.

The subtheme of family support highlights the crucial role of family involvement in the treatment journey of women diagnosed with schizophrenia. Participants specifically indicated their need for support from their families. Some expressed that they did not receive adequate support and voiced their dissatisfaction with this experience. Participants’ statements are as follows:

Participant 2: “I stayed in the hospital in Erzurum for a month during that time. I waited a long time for my family, but they did not come. None of them came. I needed their support the most.”

Participant 4: “I needed my mother the most. I love my children very much, and I needed them to be with me.”

The subtheme of the need for stigma reduction and emotional support is that women with schizophrenia must prevent stigmatization and seek acceptance from society, which is crucial and serves as a key element in facilitating access to treatment. It was found that emotional support from the surroundings of women diagnosed with schizophrenia is another factor that aids in accessing treatment. It can be said that emotional support is the second most vital element that women require for treatment access. The participants’ statements are as follows:

Participant 6: “I needed my family and those around me to treat me well. I wish they wouldn’t treat me like I am crazy.”

Participant 8: “I needed love the most. I did not receive adequate love.” (Patient 8)

Participant 4: “…perhaps if they had been more respectful to me, embraced me, and not looked down on me, I could have recovered faster. People diagnosed with schizophrenia are human too. We need them to accept us with this illness…”

The need for financial assistance subtheme highlights the necessity for financial support to cover expenses such as travel and medication to access and continue treatment. The participants’ statements are as follows:

Participant 10: “I needed money… I use a minibus to go to the hospital, to go to the CMHC, and it costs a lot of money”

Participant 4: “I need to take my medications regularly so that I do not miss my treatment, but the state charges a co-payment for the medications. It is a low fee, but it becomes a problem when I do not have money. The state should not charge for our medications…”

#### 3.1.5. Theme 5: Alternative Paths

This theme emphasizes that women diagnosed with schizophrenia not only seek medical help for recovery, but some participants also attempt to benefit from non-medical methods. However, participants indicated that they did not perceive any advantages from these non-medical approaches. This theme consisted of two subthemes such as amulet and healing and herbal treatment methods.

The amulet and healing subtheme highlights those participants employed methods such as having amulets inscribed and consulting an imam, which are prevalent in the region where the study was conducted, to alleviate their illnesses. They hoped that the imam, regarded as a religious authority in the area, would heal them through verses and prayers. Many participants turned to this approach due to family pressure. The participants’ statements are as follows:

Participant 8: “There was an Imam; I went to him first. When that didn’t work for me, I later visited a doctor.”

Participant 10: “First, my father took me to a cleric. Then my mother had an amulet made for me. My family took me…”

The herbal treatment method subtheme highlights that female schizophrenia patients often utilize various herbal products for treatment prior to seeking hospital care. The participants’ statements are as follows:

Participant 4: “When I was feeling very unwell, I tried a herbal remedy, but it didn’t help, and I got worse. Then my mother wanted to take me to the doctor, but I preferred to see a psychologist first, not a doctor.”

## 4. Discussion

This study aimed to explore the experiences of female patients with schizophrenia in accessing treatment through the lens of mental health rehabilitation services in eastern Turkey. The use of purposive sampling ensured that participants offered in-depth insights into their treatment access experiences, while reflexivity throughout the research process enhanced the reliability and rigor of the findings. By documenting assumptions and critically engaging with the data, the researchers minimized bias and facilitated a robust interpretation of the identified themes. The findings indicate that female patients with schizophrenia encounter barriers to accessing treatment, such as stigma, clinical obstacles, and access issues, and develop resistance and adaptive behaviors to cope with these challenges and their needs in seeking treatment. This study underscores significant barriers to accessing treatment while also identifying needs.

The first theme from this study, “shadow of obstacles”, emphasizes the problems experienced by female patients with schizophrenia in accessing treatment. This theme consisted of three subthemes, namely the impact of stigma, clinical barriers and access barriers. The findings from this study indicate that women diagnosed with schizophrenia face significant social stigma, which hinders their access to treatment. Furthermore, negative societal attitudes toward psychiatric medications lead these women to be influenced by their surroundings, causing them to avoid using their prescribed medications. This situation has adversely impacted the treatment processes for women diagnosed with schizophrenia. Existing literature highlights several barriers to treatment access for schizophrenia, particularly for female patients. These barriers are multifaceted, encompassing social, psychological, and systemic factors. A notable barrier is the stigma associated with mental illness, which disproportionately affects female patients. For instance, it was found that female patients reported greater treatment barriers and stigma-related obstacles compared to their male counterparts [28]. Similarly, research emphasizes how self-stigma among women can diminish their self-esteem and exacerbate the risk of poor treatment outcomes, complicating their treatment journey [29]. These findings contribute to a deeper understanding of the treatment barriers faced by female patients, building on existing literature. Unlike previous studies, this research provides a more comprehensive exploration of the pronounced effects of social stigma on women and underscores the psychological and societal dimensions of treatment access. The findings highlight the need for targeted interventions to address the unique challenges that women with schizophrenia face in accessing and adhering to treatment.

The subtheme of clinical barriers in this study highlights several significant obstacles faced by women diagnosed with schizophrenia in accessing treatment. These barriers include compulsory hospitalization, the restrictive nature of psychiatric services, stigmatizing attitudes from the treatment team, and the inability to establish effective therapeutic communication with the treatment team. These challenges contribute to delays in the initiation of treatment and hinder the overall treatment process.

Furthermore, the compulsion to remain in the hospital, which many participants found uncomfortable, was identified as an additional barrier to care. The findings indicate that women with schizophrenia are particularly troubled by compulsory hospitalization. While patients generally do not welcome involuntary admission, it is a frequent occurrence, especially during psychotic episodes, and is crucial for both the patient’s and the community’s health. Female patients, particularly those who are younger and diagnosed with psychotic disorders, are at a higher risk of compulsory hospitalization [30]. These women face unique challenges contributing to their involuntary admissions, such as societal stigma and a lack of adequate support systems. Additionally, the psychological impact of compulsory hospitalization cannot be overlooked. Involuntary admission can lead to feelings of humiliation and loss of autonomy, which may exacerbate schizophrenia symptoms and hinder recovery. This psychological distress is especially relevant for female patients, who are often already vulnerable due to societal pressures and expectations surrounding mental health. Similar findings have been reported in previous studies. Study highlights those female patients, particularly those with psychotic disorders, face an increased likelihood of compulsory hospitalization [30]. The stigmatization of mental illness and the lack of adequate mental health resources may contribute to this increased risk. Additionally, it emphasizes the psychological toll of involuntary admission, noting that feelings of humiliation and powerlessness are common experiences among patients [31]. For women, these experiences may be further compounded by societal expectations and pressures, making it more challenging for them to navigate the healthcare system and seek appropriate care. These findings emphasize the need to reconsider the approach to compulsory hospitalization for female patients with schizophrenia. The negative psychological impact of involuntary admission, particularly feelings of humiliation and loss of autonomy, warrants special attention. Previous research has shown that these factors can worsen symptoms and delay recovery [31]. To improve treatment outcomes, it is essential to address both structural barriers, such as compulsory hospitalization, and the psychological distress associated with these practices. Additionally, the findings suggest that societal stigma and inadequate support systems significantly shape these experiences, underscoring the need for comprehensive support structures and personalized treatment approaches for women with schizophrenia.

Another key factor is the perception that women are not understood by their healthcare providers. This lack of understanding delays the treatment initiation process and can result in the patients seeking alternative doctors, which further prolongs the treatment process. Research in the literature supports the notion that stigma and poor communication within healthcare settings exacerbate treatment delays and hinder access to care. Stigmatizing attitudes from healthcare providers not only affect patients’ willingness to seek treatment but also contribute to negative healthcare experiences. It was found that patients who perceive their healthcare providers as unsympathetic or dismissive are less likely to adhere to treatment plans, which in turn affects their mental health outcomes [28]. Similarly, poor communication between patients and healthcare providers often leads to feelings of marginalization and misunderstanding, further complicating the treatment process [32]. These findings underscore the critical role of addressing both structural and interpersonal barriers in improving treatment outcomes for women with schizophrenia. The restrictive nature of psychiatric services and compulsory hospitalization are significant contributors to patient discomfort and reluctance to engage in treatment. Additionally, the inability to establish therapeutic communication due to stigmatizing attitudes from healthcare providers highlights the need for improved training and awareness within clinical settings. These findings are consistent with previous research, which suggests that fostering a collaborative and non-judgmental therapeutic relationship can positively influence treatment adherence and reduce patient distress [33,34]. Moreover, addressing these barriers is essential not only for the individual patient but also for their families and caregivers, who often share the burden of navigating a challenging healthcare system [35].

The subtheme of access barriers highlights several challenges faced by women diagnosed with schizophrenia, particularly those living in villages or districts without mental health specialists. These barriers include financial constraints, such as the absence of social security, and a lack of awareness about their condition. Women in these areas face difficulties related to geographical distance, limited financial resources, and insufficient information about their illnesses. Additionally, the absence of social security complicates access to costly treatments. Another factor hindering treatment access is the lack of knowledge about their illness or uncertainty regarding which healthcare provider to consult. Furthermore, some participants reported that family members prevent them from visiting community mental health centers (CMHCs), which offer rehabilitation and treatment follow-up, posing yet another obstacle to effective treatment.

Additionally, the absence of social security adversely impacts access to costly treatment options. The intersection of socioeconomic status and gender cannot be overlooked. Women in lower socioeconomic strata are less likely to receive effective treatment compared to men, primarily due to disparities in health insurance and access to healthcare resources [36]. This socioeconomic disadvantage can exacerbate the challenges faced by female patients, leading to untreated symptoms. Moreover, addressing logistical barriers, such as transportation and appointment scheduling, is essential for improving access. Women often report difficulties in accessing services due to inconvenient locations or limited availability of appointments [37]. By implementing flexible scheduling and providing transportation assistance, healthcare systems can significantly enhance treatment access for female patients.

Similar barriers to treatment access have been reported in other studies, with socioeconomic factors and geographical distance playing a significant role. Research highlights how women in lower socioeconomic strata are less likely to receive effective treatment compared to their male counterparts, primarily due to disparities in health insurance and access to healthcare resources [36]. This socioeconomic disadvantage is often compounded by logistical barriers, such as the lack of affordable transportation and difficulties with appointment scheduling. Studies have shown that women, particularly those in rural areas, report considerable challenges in accessing healthcare services due to the inconvenient locations of treatment centers and the limited availability of appointments [37]. These findings underscore the intersection of socioeconomic status and gender, which significantly impacts access to care for women with schizophrenia. The absence of social security and financial constraints exacerbate these challenges, leading to untreated symptoms and worse outcomes for female patients. Addressing these barriers requires a multifaceted approach that includes improving the availability of mental health services in underserved areas, providing financial support through social security or alternative health insurance options, and ensuring logistical support such as transportation and flexible appointment scheduling. These measures can help reduce the disparities in treatment access between men and women and improve overall treatment adherence and outcomes for female patients.

The theme from the study, “resistance and adaptation”, highlights the positive responses participants made in coping with the challenges they faced, such as relocating to facilitate easier access to treatment and demonstrating a strong commitment to recovery. It also addresses negative responses, such as stopping medication and being unable to leave the house due to the impact of stigma.

One of the prominent findings is that many participants demonstrated a strong commitment to recovery by actively seeking solutions to overcome barriers to treatment access. This included relocating to areas with better access to mental health services, which reflects a proactive approach to managing their condition. Such mobility is crucial as it not only facilitates treatment adherence but also enhances the overall quality of life for these women [38]. The importance of environmental factors in influencing treatment outcomes has been documented, emphasizing that reduced accessibility can lead to poorer functional outcomes in patients with schizophrenia [38]. This aligns with the notion that overcoming physical barriers can significantly improve engagement with healthcare services. Conversely, the study also identified negative responses among participants, particularly the tendency to withdraw from treatment due to stigma and social isolation. Many women reported feeling stigmatized, which led to discontinuing medication and remaining homebound. This withdrawal can exacerbate their condition, as social isolation is known to correlate with worse clinical outcomes in schizophrenia [39]. The impact of stigma on treatment adherence is well-documented, indicating that negative societal perceptions can deter individuals from seeking necessary care. Moreover, the psychological stress associated with stigma can further hinder recovery efforts, leading to a cycle of disengagement from treatment [40].

The subthemes of autonomy and social isolation are particularly noteworthy. While some participants expressed a desire for autonomy in managing their treatment, this often conflicted with their experiences of social isolation. The struggle for autonomy can be empowering; however, when combined with feelings of isolation, it can lead to detrimental outcomes such as decreased motivation and increased reliance on maladaptive coping strategies [41]. The duality of seeking independence while grappling with social withdrawal underscores the need for tailored interventions that address both the psychological and social dimensions of recovery in female patients with schizophrenia [42]. In summary, the results of this study illuminate the nuanced experiences of female patients with schizophrenia in accessing treatment. Positive responses, such as relocating for better access, demonstrate resilience and commitment to recovery. However, negative responses, including withdrawal and social isolation due to stigma, highlight significant barriers that need to be addressed. These findings underscore the importance of developing comprehensive support systems that not only facilitate access to treatment but also combat stigma and promote social inclusion.

Another important theme from the study, “Connection and Solidarity”, emphasizes the critical role of both individual and institutional factors in facilitating effective treatment. The subthemes of self-motivation, family support, and support from healthcare institutions each play an important role in shaping the treatment experiences of women with schizophrenia. Self-motivation is a crucial individual factor that influences treatment adherence and overall mental health outcomes. Research indicates that individuals who possess a strong sense of autonomy and internal motivation are more likely to engage in health-promoting behaviors and adhere to treatment regimens. This aligns with findings from studies that emphasize the importance of self-determination in fostering psychological well-being and maintaining behavioral changes post-treatment [43]. In the context of schizophrenia, self-motivation can be particularly pivotal, as it empowers women to actively participate in their treatment journey, seek help, and adhere to prescribed therapies. Family support emerges as another vital component in the treatment landscape for women with schizophrenia. Studies have shown that high levels of family support correlate with improved quality of life and better social functioning among patients [44,45]. Specifically, emotional, instrumental, and informational support from family members can significantly enhance treatment adherence and reduce the stigma associated with mental illness [44]. The presence of a supportive family environment not only fosters a sense of belonging but also mitigates feelings of isolation that often accompany schizophrenia [46]. Furthermore, family involvement in treatment planning and decision-making processes can lead to more favorable outcomes, as it aligns treatment goals with the patient’s personal values and needs [47].

Institutional support, including access to community mental health centers and free healthcare services, is essential for women seeking treatment for schizophrenia. In Turkey, community mental health centers (CMHC) aim to provide effective treatments designed to enhance individual functions within the community-based mental health model. Treatment and monitoring are conducted using a multidimensional psychosocial approach, ensuring that care needs are met [48,49]. Ensari et al. (2013) reported that CMHC practices are beneficial in improving the quality of life, reducing disability, and increasing functionality in patients with schizophrenia [50]. It was also found that significant improvements in both clinical and social functionality measures when comparing the initial and third-year assessments [51]. Additionally, 22.9% of the CMHC sample resided in nursing homes, with many lacking incomes and coming from low socioeconomic backgrounds [52]. It was emphasized that a patient’s functionality, independence, and income status are crucial factors concerning family and caregiver burden [51]. The availability of resources such as community mental health centers can provide critical support in terms of therapy, medication management, and social services [47]. Access to these services is often hindered by systemic barriers, including stigma, high treatment costs, and limited availability of mental health professionals [53]. Therefore, ensuring that women have free and equitable access to mental health care is fundamental to improving treatment outcomes. Studies indicate that when healthcare institutions actively promote a supportive environment and facilitate access to care, patients are more likely to engage in treatment and experience positive health outcomes [39]. In conclusion, the theme of “Connection and Solidarity” highlights the interplay between individual motivation, family dynamics, and institutional support in the treatment of women with schizophrenia. By fostering self-motivation, enhancing family support, and ensuring robust institutional resources, mental health care can be significantly improved for this population. These findings underscore the necessity for a holistic approach that integrates personal, familial, and systemic factors to optimize treatment experiences and outcomes for women with schizophrenia.

Another theme from the study, “unmet needs”, emphasizes the basic needs of women diagnosed with schizophrenia, such as the need for family support in obtaining and maintaining treatment, the need for stigma reduction and emotional support, and the need for financial assistance. Women diagnosed with schizophrenia face significant challenges in accessing and sustaining treatment, as these unmet needs directly impact their ability to seek and adhere to treatment. The lack of family support can hinder their treatment engagement and recovery. Additionally, the stigma associated with schizophrenia exacerbates the emotional burden of these women, further complicating their mental health and treatment journey. Financial difficulties, including the inability to afford treatment and related expenses, pose another substantial barrier to effective care.

Existing literature supports the idea that unmet needs are crucial in determining the treatment outcomes of female patients with schizophrenia. Studies have shown that family support is a key factor in treatment adherence and recovery. Women with strong family support networks are more likely to engage in treatment and experience better outcomes [54]. In contrast, the lack of family support often leads to isolation, worsening symptoms, and limited access to care. Additionally, the stigma surrounding schizophrenia has been identified as a significant barrier to treatment. Research highlights the emotional toll of stigma, which discourages women from seeking help and negatively impacts their self-esteem and willingness to adhere to treatment [55,56]. Financial difficulties also serve as a barrier to accessing healthcare, with many women unable to afford the necessary treatment and medications [57]. These findings emphasize the multifaceted nature of unmet needs, which extend beyond clinical care to include emotional, social, and financial aspects. Previous studies underscore the importance of family support, stigma reduction, and financial assistance, advocating for a holistic, integrated approach to care. Addressing these needs not only improves treatment adherence but also enhances the overall well-being of female patients. The findings stress the importance of healthcare systems recognizing and addressing these unmet needs to improve treatment outcomes. Strategies such as family-centered care, anti-stigma campaigns, and financial assistance programs could significantly improve access to care and recovery for women with schizophrenia.

The treatment barriers faced by female patients with schizophrenia are multifaceted and closely linked to their social roles and societal context. A significant barrier identified in the present study was obstruction from families, preventing access to Community Mental Health Centers (CMHCs). This barrier can be understood in the context of the social roles ascribed to women within the cultural framework of the region. Research indicates that women with schizophrenia face unique social challenges compared to men, which can hinder access to care and affect treatment outcomes. Female patients are disproportionately affected by social risk factors such as childhood and adult trauma, victimization, stigma, and socioeconomic disadvantages, leading to more severe mental health issues and worse treatment outcomes compared to men [54]. This gender disparity underscores the need for gender-sensitive approaches to schizophrenia treatment. Societal expectations related to motherhood further complicate the treatment process for women with schizophrenia. The inability to fulfill maternal roles often leads to feelings of inadequacy, compounded by social stigma surrounding mental illness. These feelings not only intensify the emotional burden of schizophrenia but also create additional barriers to treatment. Studies show that societal pressures significantly impact mental health treatment and recovery for women [55]. The complex interplay between social factors and schizophrenia symptoms in women requires treatment plans tailored to their specific needs and challenges [56]. Another crucial factor influencing treatment outcomes is social support. It has been consistently shown that strong social support plays a vital role in treatment acceptance and adherence. Women with schizophrenia who receive robust social support are more likely to engage in treatment and experience better recovery outcomes. Interventions that enhance social networks and support systems could, therefore, be beneficial for women facing significant treatment barriers [57]. In conclusion, the treatment barriers encountered by women with schizophrenia are deeply rooted in their social roles and the unique challenges they face. These challenges include societal expectations surrounding motherhood, the impact of trauma and stigma, and the need for robust social support networks. To improve outcomes for female patients with schizophrenia, it is crucial to address these barriers through gender-sensitive treatment approaches and by enhancing social support structures. Such interventions are essential for improving treatment access, adherence, and overall mental health outcomes for women diagnosed with schizophrenia.

The current study highlights that some women diagnosed with schizophrenia explore “alternative paths”, such as spiritual approaches, in addition to seeking medical treatment. Specifically, participants in this study reported engaging in practices such as having amulets inscribed and consulting a *hodja* (spiritual healer) as prevalent in the cultural context of the study area. These women hoped that the *hodja*, regarded as a religious authority, could alleviate their symptoms through prayers and verses. However, participants indicated that they did not perceive any significant benefits from these non-medical approaches, suggesting a gap between their expectations and the outcomes of such practices. Existing literature has explored the role of non-medical and spiritual treatment methods among individuals with schizophrenia, particularly in Muslim communities. A study by Shanko et al. (2023) found that 88% of participants in Ethiopia preferred spiritual or traditional healing methods for schizophrenia, highlighting the prominent role of spirituality in healthcare decisions within certain cultural contexts [58]. Similar findings are reported by Boti et al. (2020), who emphasize that spiritual practices are often perceived as more culturally acceptable and accessible compared to conventional psychiatric care [59]. This aligns with the current study, where women in the sample sought spiritual healing methods due to cultural familiarity and the sense of belonging, they provided. The reliance on spiritual approaches to managing schizophrenia can provide emotional and social support, which is vital in alleviating the stigma surrounding mental illness. Research has shown that spirituality can enhance coping strategies and contribute to a sense of community, which is particularly important for individuals who face societal exclusion due to mental health issues [60,61]. Furthermore, spiritual practices may improve the overall quality of life for individuals with schizophrenia, even if they do not directly address the symptoms of the disorder [62]. In this sense, the study supports the notion that spirituality plays a supportive role in the lives of patients, offering emotional comfort.

However, a critical concern with the reliance on non-medical approaches is the potential delay in seeking effective psychiatric care. The prioritization of spiritual treatments over evidence-based medical care can result in worsening symptoms and increased relapse risks. Moreover, spiritual practices do not address the biological and psychological aspects of schizophrenia, which are crucial for successful recovery and management [61,63,64]. This gap between cultural practices and the medical needs of patients underscores the importance of integrating medical and cultural interventions in the treatment process. Furthermore, the role of spiritual healers in the treatment of schizophrenia must be carefully considered. As Khattak et al. (2022) argue, while spiritual healers are respected within their communities, they may lack the necessary medical expertise to treat the complexities of mental illness. This can lead to inappropriate or even harmful interventions [65]. Additionally, the emphasis on spiritual causes of mental illness can reinforce stigma, potentially contributing to the perception of mental health conditions as moral or spiritual failures, rather than medical conditions that require treatment [61].

The findings from this study, in conjunction with existing literature, suggest that while spiritual practices such as consulting a *hodja* offer emotional support and community solidarity for women with schizophrenia, they present significant risks when they replace or delay conventional psychiatric care. The reliance on these methods may exacerbate symptoms and hinder recovery if medical treatment is neglected. It is essential for healthcare providers to recognize the cultural importance of spiritual practices within Muslim communities while advocating for a balanced approach that integrates both medical and spiritual interventions. This approach would ensure that women with schizophrenia receive comprehensive care, addressing both their cultural needs and their medical conditions, thereby improving their overall treatment outcomes.

## 5. Conclusions

In conclusion, this qualitative study explored the experiences of female patients with schizophrenia in Eastern Turkey and highlighted several critical themes related to their access to treatment. The findings underscore the importance of understanding gender-specific factors that influence treatment adherence and health outcomes. Female patients often face unique challenges, including societal stigma, gender-based discrimination, and familial influences that hinder their treatment progress. The quality of care provided by healthcare professionals also varied significantly, with many women expressing dissatisfaction due to a lack of understanding and empathy from mental health services. This dissatisfaction often led to a further reluctance in seeking necessary treatment.

The study also emphasized the crucial role of community-based mental health care centers, where female patients can receive essential support and resources. The need for more comprehensive services that address both the medical and psychosocial aspects of schizophrenia was also evident. Additionally, it became clear that gender-sensitive treatment approaches, tailored to the specific experiences and challenges of female patients, would improve treatment outcomes.

Despite the valuable insights provided, this study has several limitations. The small sample size may limit the generalizability of the findings to a larger population. While qualitative research aims to provide in-depth understanding, rather than statistical generalization, future research could include a larger sample to enhance external validity. Moreover, participants with comorbid conditions were excluded to maintain homogeneity in the sample, which may have limited the diversity of the experiences captured in the study. Including individuals with multiple conditions in future research would provide a broader understanding of the challenges faced by patients with complex health needs. Another potential limitation is interviewer bias, as researchers may unintentionally influence participants’ responses. To address this, a reflexive approach was adopted, and multiple researchers were involved in the coding process to enhance the objectivity of the analysis. Additionally, member checking was conducted to ensure the accuracy of interpretations. Finally, the findings are based on data collected in a specific cultural and regional context, which may limit their applicability to other settings. Future studies should explore similar phenomena in different cultural and geographical contexts to understand potential variations in experiences and treatment outcomes.

Future research should focus on developing and implementing interventions that are specifically tailored to the unique needs of female patients with schizophrenia. These interventions should incorporate gender-sensitive strategies to ensure equitable access to care and support. Additionally, ongoing training for healthcare providers regarding gender dynamics in mental health treatment could significantly improve the quality of care for female patients. It is also essential to explore the integration of both medical and spiritual treatments, especially in communities where non-medical approaches are prevalent. Understanding how these practices impact treatment adherence and outcomes will be crucial in designing comprehensive care models. Furthermore, research examining the role of social support systems, including family and community involvement, in improving treatment access and outcomes for female patients would be beneficial.

In conclusion, this study provides valuable insights into the barriers faced by female patients with schizophrenia in Turkey and the need for a more nuanced understanding of their treatment experiences. Addressing these barriers through targeted interventions, increased awareness, and the integration of gender-sensitive approaches in mental health care will lead to improved treatment experiences and outcomes for women living with schizophrenia.

## Figures and Tables

**Table 1:** Demographic characteristics of participants (n = 10).

**Characteristic**	**Category**	**Number of Participants**	**Percentage (%)**
Marital Status	Married	3	30
	Single	7	70
Employee status	Yes	0	0
	No	10	100
Income Status	Poor	8	80
	Middle	2	20
	High	0	0
History of psychotic illness in the family	Yes	1	10
	No	9	90
**Characteristic**		**Mean**	**Min-Max**
Age		41.40	25–51
Length of disease (years)		21.20	5–37

## Data Availability

Data supporting the findings of this study are available within the paper; the rest of the data that support the findings of this study are not openly available due to reasons of sensitivity and are available from the corresponding author upon reasonable request.
